# Supplementary material for: Cannabidiol changes P-gp and BCRP expression in trophoblast cell lines
Source: PeerJ. 2013 Sep 12;1:e153. doi: 10.7717/peerj.153 (PMC3775628; doi:10.7717/peerj.153)
Supplement: Table S1 [file peerj-01-153-s001.doc]

**Cannabidiol changes P-gp and BCRP expression in trophoblast cell lines.**

V. Feinshtein, O. Erez, Z. Ben-Zvi, T. Eshkoli, B. Sheizaf, E. Sheiner, M. Huleihel, G. Holcberg.

**PeerJ**

**Supp.1: Summarizing table of all antibodies used in the research.**

| **Antibody** | **Manufacturer** | **Catalog number, Dilution** |
| --- | --- | --- |
| Rabbit monoclonal anti-Na^+^/K^+^ ATPase antibody | Abcam (Cambridge, UK) | ab76020, 1:1000 |
| Mouse monoclonal anti-actin antibody | MP Biomedicals (Solon, OH) | 691001, 1:50000 |
| Rabbit polyclonal anti-NF-kB p65 antibody | Santa Cruz Biotechnology (Santa Cruz, CA) | sc-372, 1:100 |
| Mouse anti-ABCG2 (BXP-21) | Santa Cruz Biotechnology (Santa Cruz, CA) | sc-58222, 1:100 |
| Mouse monoclonal anti-P-glycoprotein C219 (preferentially recognizes the MDR1 isoforms of Pgp) | Calbiochem (San Diego, CA) | 517310, 1:55 |
| Mouse anti-human CD 243 (MDR-1) antibody | BioLegend (San Diego, CA) | clone UIC2, 348601, 1:50 |
| AlexaFluor-488 goat anti-mouse antibody | Jackson ImmunoResearch Laboratories (West Grove, PA) | 115-545-146, 1:100 |
| mouse anti human anti ABCG2 FITC conjugated, clone 5D3 | Chemicon (Millipore, Merck KGaA, Darmstadt, Germany) | MAB4155F, 1:10 |
| HRP-conjugated donkey anti-rabbit antibody | Amersham (Piscataway, NJ) | NA934 |
| HRP-conjugated goat anti-mouse antibody | Jackson ImmunoResearch Laboratories (West Grove, PA) | 115-035-062 |
